# Giant unruptured middle cerebral artery aneurysm revealed by intracranial hypertension: is a systematic decompressive hemicraniotomy mandatory?

**DOI:** 10.1007/s10143-024-02662-z

**Published:** 2024-08-09

**Authors:** Rabih Aboukais, Antoine Devalckeneer, Pierre Boussemart, Philippe Bourgeois, Tomas Menovsky, Martin Bretzner, Mélodie-Anne Karnoub, Jean-Paul Lejeune

**Affiliations:** 1 Department of Neurosurgery, Lille University Hospital, rue E. Laine, Lille cedex, 59037 France; 2 University Lille, INSERM, CHU Lille, U1189-ONCO-THAI-Image Assisted Laser Therapy for Oncology, Lille, F-59000 France; 3 Neurosurgical Intensive Care Department, Lille University Hospital, Lille, France; 4 Department of Neurosurgery, Antwerp University Hospital, University of Antwerp, Antwerp, Belgium; 5 Department of Neuroradiology, Lille University Hospital, Lille, France

**Keywords:** Giant aneurysm, Microsurgery, Mca, Bypass, Anastomosis, Deep hypothermic circulatory flow arrest

## Abstract

Our study aimed to evaluate the postoperative outcome of patients with unruptured giant middle cerebral artery (MCA) aneurysm revealed by intracranial hypertension associated to midline brain shift. From 2012 to 2022, among the 954 patients treated by a microsurgical procedure for an intracranial aneurysm, our study included 9 consecutive patients with giant MCA aneurysm associated to intracranial hypertension with a midline brain shift. Deep hypothermic circulatory flow reduction (DHCFR) with vascular reconstruction was performed in 4 patients and cerebral revascularization with aneurysm trapping was the therapeutic strategy in 5 patients. Early (< 7 days) and long term clinical and radiological monitoring was done. Good functional outcome was considered as mRS score ≤ 2 at 3 months. The mean age at treatment was 44 yo (ranged from 17 to 70 yo). The mean maximal diameter of the aneurysm was 49 mm (ranged from 33 to 70 mm). The mean midline brain shift was 8.6 mm (ranged from 5 to 13 mm). Distal MCA territory hypoperfusion was noted in 6 patients. Diffuse postoperative cerebral edema occurred in the 9 patients with a mean delay of 59 h and conducted to a postoperative neurological deterioration in 7 of them. Postoperative death was noted in 3 patients. Among the 6 survivors, early postoperative decompressive hemicraniotomy was required in 4 patients. Good functional outcome was noted in 4 patients. Complete aneurysm occlusion was noted in each patient at last follow-up. We suggest to discuss a systematic decompressive hemicraniotomy at the end of the surgical procedure and/or a partial temporal lobe resection at its beginning to reduce the consequences of the edema reaction and to improve the postoperative outcome of this specific subgroup of patients. A better intraoperative assessment of the blood flow might also reduce the occurrence of the reperfusion syndrome.

## Introduction

Unruptured giant intracranial aneurysms (≥ 25 mm) have a poor natural history with mortality rates of 68% and 80% over 2-year and 5-year without treatment, respectively [9, 17]. Intracranial hemorrhage and thrombo-embolic ischemic lesions are the two mechanisms responsible for this high rate of morbimortality [11, 23]. Deep hypothermic circulatory arrest/ extra-corporeal circulation with vascular reconstruction [19, 20] and/or cerebral revascularization with parent artery sacrifice are usually the two main surgical procedures to exclude the giant aneurysmal sac from the circulation. The morbidity and the mortality of these treatments remain too high in this context, between 10 and 60% and 4 and 10% [13, 15, 20, 29]. respectively. The morbimortality rate is slightly reduced with endovascular treatment but with a higher risk of recanalization [8, 23]. Nevertheless, in our institution, we identified a subgroup of patients with an unruptured giant middle cerebral artery aneurysm (MCA) revealed by intracranial hypertension associated to a midline brain shift, in which the postoperative outcome appeared more severe than other subgroups of patients with an unruptured giant anterior circulation aneurysm. Indeed, we observed that an early postoperative decompressive hemicraniotomy (DH) was always required in this specific subgroup of patients because of a rapid neurological deterioration occurrence related to a significant postoperative edema reaction.

Our study aimed to evaluate the postoperative outcome of 9 consecutive patients with an unruptured giant MCA aneurysm revealed by intracranial hypertension associated to a midline brain shift.

## Methods

### Population data

In our institution, treatment (endovascular or microsurgical) was decided in a multidisciplinary discussion between neurosurgeons, neuroradiologists and intensivists.

From 2012 to 2022, among the 3128 patients with ruptured or unruptured intracranial aneurysm managed in our institution, 954 patients were treated by a microsurgical procedure. Of these 954 patients, our study included 9 consecutive patients who had an unruptured giant MCA aneurysm associated to intracranial hypertension with a midline brain shift. Patients with a ruptured giant aneurysm or patients with a large/complex aneurysm (< 25 mm) were both excluded from this study. Patients without a midline brain shift were also excluded.

### Pretherapeutic clinical evaluation

Age at treatment, medical history and the initial neurological presentation of the aneurysm were recorded. Funduscopic examination was performed to detect papilledema related to intracranial hypertension.

### Pretherapeutic radiological evaluation

The size and location (M1, MCA bifurcation…) of the aneurysm as well the angioarchitecture were evaluated on a conventional cerebral angiography and on a magnetic resonance imaging (MRI). The presence of a peri-aneurysmal brain edema and midline brain shift were also evaluated on MRI. Calcifications of aneurysm wall and intrasaccular thrombus were documented using cranial computed tomography (CT) scanning and MRI. Perfusion CT scan was done to detect any cerebral hypoperfusion related to a potential flow steal related to the giant aneurysm.

When a bypass was proposed in the surgical strategy, the quality of STA branches was assessed on the conventional external carotid angiogram. Venous Doppler and cervical Doppler allowed the evaluation of vascular graft quality (ulnar vein, saphenous vein, radial artery) as well as the evaluation of the internal carotid artery (ICA) and the external carotid artery (ECA), if a high flow bypass was planned in the surgical strategy.

When an extracorporeal circulation was needed, a cardio-vascular assessment and a discussion with the cardio-vascular surgical team were performed.

### Treatment

Deep hypothermic circulatory flow reduction (DHCFR) with extracorporeal circulation was the first surgical procedure that we performed in our institution to treat young patients with a giant aneurysm. A significant morbidity (thrombo-embolic ischemic lesions) related to the aneurysmal neck and sac surgical manipulation was noted. Ischemic lesions were also due to the intra/postoperative reversion of the deep hypothermia (prothrombotic factors). To reduce this morbidity to the DHCFR (coagulopathy, ischemic and/or hemorrhagic stroke), we have progressively reoriented our therapeutic strategy towards cerebral revascularization using bypass surgery before aneurysm surgical trapping. DHCFR with vascular reconstruction was done in 4 patients. Bypass associated with aneurysm trapping was the therapeutic strategy in 5 patients. Intraoperative doppler and indocyanine green videoangiography were performed during the procedures to confirm aneurysm exclusion, patency of the vascular anastomosis and patency of all arterial vessels.

### Postoperative evaluation

Repetitive clinical and neurological examination were performed by a senior neurosurgeon and a senior intensivist immediately after the surgical procedure. Early transcranial Doppler, MRI, CT scan, and conventional angiography were performed in each patient to confirm the patency of bypasses, to measure flow in the anastomoses, to detect ischemic lesions, and to evaluate the exclusion of the aneurysm. All patients were re-evaluated three months after treatment. Clinical and neurological examination and the modified Rankin Scale score (mRS) were recorded. Late postoperative (> three months) MR imaging was performed in each patient to detect any ischemic lesions and to evaluate cerebral perfusion. A late conventional angiography was performed after 1 year to evaluate the occlusion of the aneurysm and the patency of the bypasses. Cranial Doppler was performed in each patient 3 months later to evaluate the flow in the anastomoses. CT angioscan was performed each year to detect any aneurysm recanalization.

## Results

### Population data

The mean age at treatment was 44 yo (ranged from 17 to 70 yo). The mean follow-up was 75 months (ranged from 0.3 to 156 months). Arterial hypertension was noted in 3 patients and 4 patients were smokers. Headache was present in 8 patients, cognitive disorder in 2 patients, motor language disorder in 1 patient and hemiparesis in 1 patient. Seizures were present in 2 patients. Visual disturbance related to papilledema was recorded in 5 patients. The mean maximal diameter of the aneurysm was 49 mm (ranged from 33 to 70 mm). Peri-aneurysmal cerebral edema was noted in 7 patients. Intrasaccular thrombus was recorded in 8 patients and wall calcifications were recorded in 5 patients. The mean midline brain shift was 8.6 mm (ranged from 5 to 13 mm) (Table 1). Distal MCA territory hypoperfusion was noted in 6 patients.

**Table 1:** Population data

Patient/Gender	Age (yo)	Maximal diameter of the MCA aneurysm (mm)	Peri-aneurysmal cerebral edema	Intrasaccular thrombus	Aneurysmal wall calcifications	Midline brain shift (mm)
1/Female	41	54	No	No	No	9
2/Male	66	60	Yes	Yes	Yes	13
3/Male	23	37	Yes	Yes	Yes	9
4/Male	70	45	Yes	Yes	No	9
5/Female	57	46	Yes	Yes	Yes	6
6/Female	44	40	Yes	Yes	No	10
7/Female	36	42	Yes	Yes	Yes	6
8/Female	17	70	No	Yes	No	11
9/Female	44	50	Yes	Yes	Yes	5

### Treatment

Preoperative corticotherapy was done in the 9 patients.

### DHCFR with extracorporeal circulation

DHCFR with vascular reconstruction and aneurysmal exclusion was performed in **Patients 3, 5, 6 and 7**. A deliberate partial temporal lobe resection was performed in **Patient 6** at the beginning of the surgical procedure to gain a better exposure of the aneurysm.

### Bypass surgery and aneurysmal trapping

**Fig. 1:**
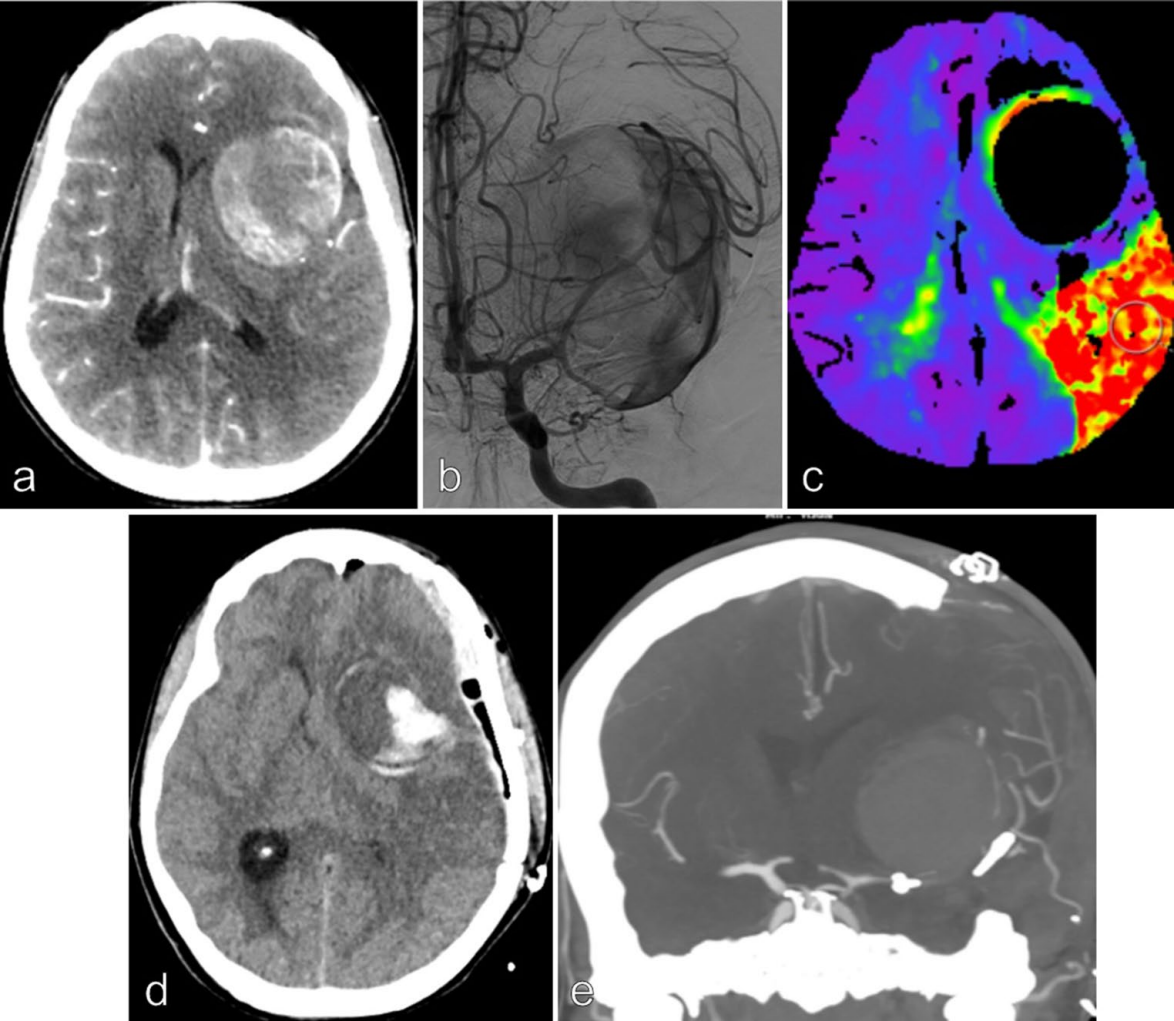
Patient 1, 41 yo female, smoker with a medical history of migraine, presented with headache and a 1-month history of moderate motor language disorder. CT scan, MRI/MRA and conventional angiography demonstrated a giant left MCA aneurysm injected only by the left temporal M2 segment. No significant edema, intrasaccular thrombus or wall calcifications were noted (**a** and **b**). The midline brain shift was 9 mm. A severe hypoperfusion in the left distal MCA territory was noted on the perfusion CT scan (**c**). A distal STA-MCA bypass was performed, followed by aneurysmal trapping and partial resection of the aneurysm sac. The patient was extubated after 24 h without any neurological deficit. 36 h after treatment, a rapid consciousness disorder with a left mydriasis occurred, related to a left hemispheric diffuse edema (**d**). A decompressive hemicraniectomy associated to a partial anterior temporal lobe resection was immediately done (**e**). A severe vasospasm appeared 5 days later and was partially improved by chemical angioplasty. The bypass was always patent. The patient improved, with a moderate language disorder but no motor deficit. The mRS score was 2 after 3 months

STA-frontal M3 segment anastomosis followed by an aneurysmal trapping was performed in **Patient 1** (Fig. 1).

**Fig. 2:**
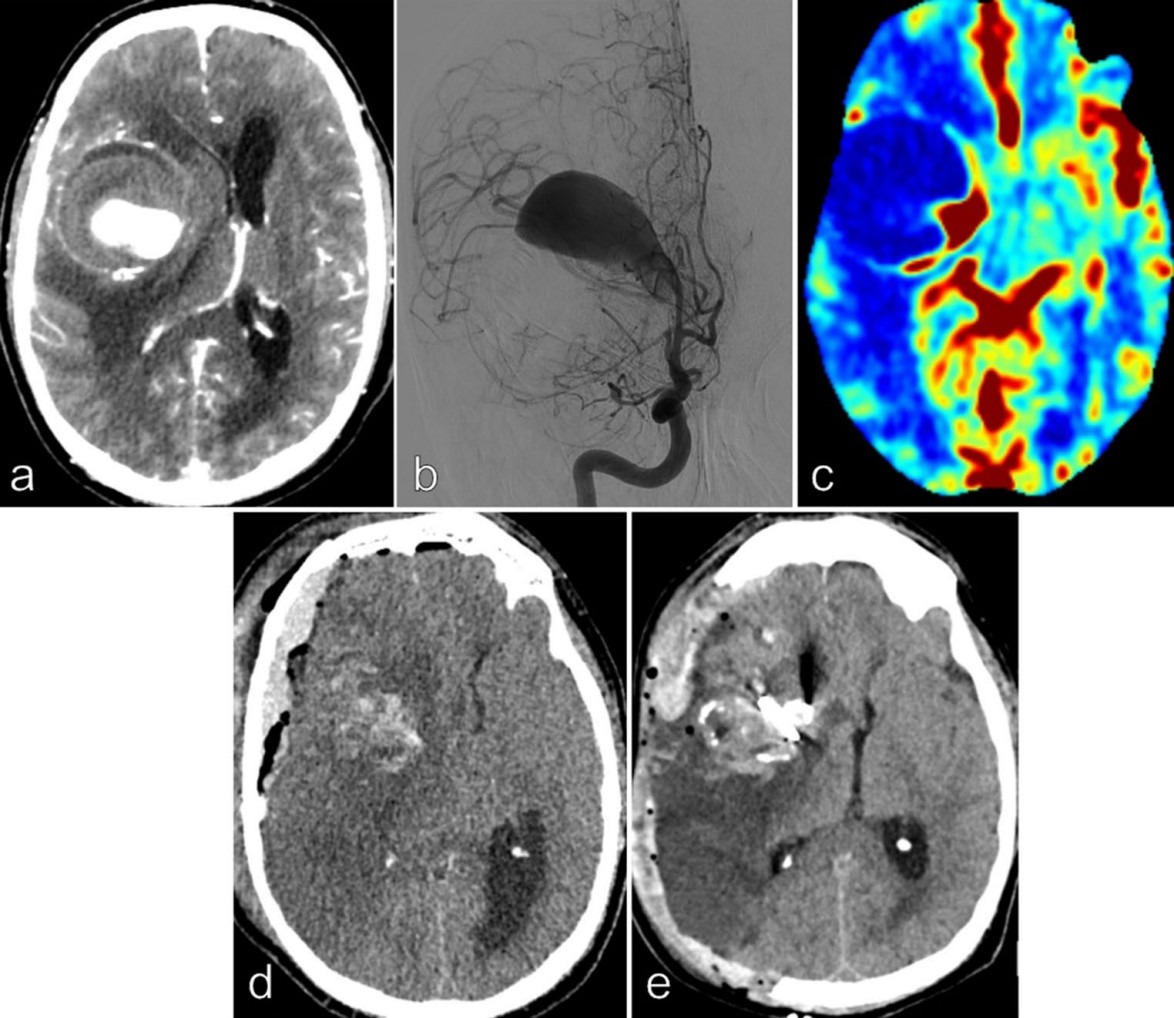
Patient 2, **66 yo** male, smoker with a medical history of chronic bronchitis and peripheral arterial occlusive disease of the lower limbs, presented with headache and a 3 weeks history of left moderate hemiparesis. CT scan, MRI/MRA and conventional angiography demonstrated a right giant MCA aneurysm (maximal diameter = 70 mm) injected only by the M1 segment. A significant peri-aneurysmal edema, wall calcifications and intrasaccular thrombus were noted. The midline brain shift was 13 mm (**a** and **b**). A severe hypoperfusion in the left distal MCA territory was noted on the perfusion CT scan (**c**). Double barrel STA-MCA bypass (on the 2 M2 segments) was done, followed by aneurysmal trapping and a partial resection of the sac and thrombus. Twelve hours after treatment, a rapid consciousness disorder with a right mydriasis occurred, related to a right hemispheric diffuse edema and ischemic lesions (**d**). Decompressive hemicraniectomy was immediately done (**e**). Fatal evolution with lung infection and scar necrosis were noted. He died 21 days after the treatment

Double barrel STA-M2 segments anastomosis followed by an aneurysmal trapping was performed in **Patient 2** (Fig. 2).

Protective STA-M4 anastomosis associated to high flow bypass (ECA-Ulnar vein-M2 segment) followed by an aneurysmal trapping was performed in **Patient 4**.

Only distal STA-MCA anastomosis was performed in **Patient 8** without aneurysm exclusion. Aneurysmal exclusion was planned in a second surgical procedure in this last patient, but intracranial hypertension dramatically worsened soon after the first procedure and the patient died.

STA-frontal M3 segment anastomosis followed by an aneurysmal trapping was performed in **Patient 9.**

For 8 patients, the giant aneurysm was completely occluded, all anastomoses and all arterial vessels were patent on the indocyanine green videoangiography at the end of the procedure.

In all these patients, the aneurysm was partially resected after partial thrombectomy.

### Flow assisted surgical techniques

The flow was measured using the intraoperative doppler in **Patient 1, Patient 2 and Patient 9**.

#### Patient 1

Double barrel STA-MCA bypass (on the 2 M3 segments) with aneurysmal trapping was decided. The mean cut flow of each STA branches was 25 mL/minute (frontal) and 19 mL/minute (parietal), respectively. Before the anastomosis, the flow measured in the 2 M3 segments was 5 mL/minute (superior segment) and 3 mL/minute (inferior segment) respectively. Finally, a unique STA-MCA bypass was performed because the inferior M3 segment was too small and only irrigated the anterior and inferior temporal zone. The flow was 20 mL/minute in the STA-superior M3 anastomosis. Aneurysmal trapping followed by a partial resection of the aneurysm sac was successfully done.

#### Patient 2

Double barrel STA-MCA bypass (on the 2 M2 segments) with aneurysmal trapping was done. The mean cut flow of each STA branches was 15 mL/minute (frontal) and 12 mL/minute (parietal), respectively. Before the anastomosis, the flow measured in the 2 M2 segments was 6 mL/minute (superior) and 5 mL/minute (inferior) respectively. The mean flow was 7 mL/minute and 6 mL/minute in the STA-MCA anastomoses, respectively. Aneurysmal trapping followed by a partial resection of the aneurysm sac and the thrombus was successfully done.

#### Patient 9

A large craniotomy was done at the beginning of the procedure (decompressive hemicraniectomy). STA-MCA bypass on the M3 frontal segment associated with an aneurysmal trapping was performed. The mean cut flow of frontal STA branch was 7 mL/minute. The mean flow measured on the M3 frontal segment (unique distal branch for revascularization) was 15 mL/minute before temporary clipping and − 7mL after temporary clipping (due to a retrograde flow from distal pial anastomosis). The mean flow was 13mL/minute in the STA-MCA (M3) anastomosis at the end of the procedure. Aneurysmal trapping followed by a partial resection of the aneurysm sac and thrombus was successfully done. The bone flap was sent to cryopreservation.

### Outcome

The 9 patients presented a diffuse postoperative edema (Table 2). Among them, 7 had a postoperative neurological deterioration. The delay between treatment and the neurological deterioration was 59 h (ranged 6 from to 240 h). Postoperative death was noted in 3 patients (< 21 days after treatment). In 2 of these 3 patients **(Patient 4 and Patient 8)**, DH was suitable but the neurological deterioration occurred very rapidly and the clinical status was judged too severe to perform this surgical procedure. The last patient **(Patient 2)** died from a diffuse infection in the intensive care unit. In these 3 patients, the anastomosis was always patent.

**Table 2:** Postoperative outcome of patients. (*: Extra Dural Hematoma)

Patient	Postoperative ischemic lesions	Postoperative neurological status	Delay for decompressive hemicraniotomy	Delay for elimination of midline brainshift	Neurological sequelae	mRS 3 months after treatment
1	No	24 h: No deficit, extubated 36 h: left mydriasis	36 h	2 months	Moderate motor language disorder	2
2	Large	24 h: Right Mydriasis	24 h	Partial regression	1 month: death from diffuse infection	6
3	No	24 h: No deficit, extubated 48 h: Left mydriasis	48 h	3 weeks	Moderate motor language disorder	2
4	Large	6 h: EDH* evacuation 24 h: Right mydriasis	24 h: No decompressive hemicraniectomy but corticotherapy	Not Applicable	21 days: Death from digestive hemorrhage	6
5	Small	24 h: Right mydriasis	24 h	2 months	Severe neurocognitive disorder and left hemiplegia	4
6	No	48 h: No deficit, extubated	Not applicable	Not Applicable	No deficit Asthenia	1
7	Large	16 h: consciousness disorder	16 h	3 months	Motor language disorder and right hemiplegia	4
8	No	10 days: consciousness disorder and right mydriasis	10 days: No decompressive hemicraniectomy Diffuse cerebral edema and partial aneurysmal thrombosis	Not Applicable	Death	6
9	No	24 h: Regressive right motor deficit, extubated	Deliberate decompressive hemicraniectomy at the end of the surgical procedure	2 months	No deficit Asthenia	1

**Fig. 3:**
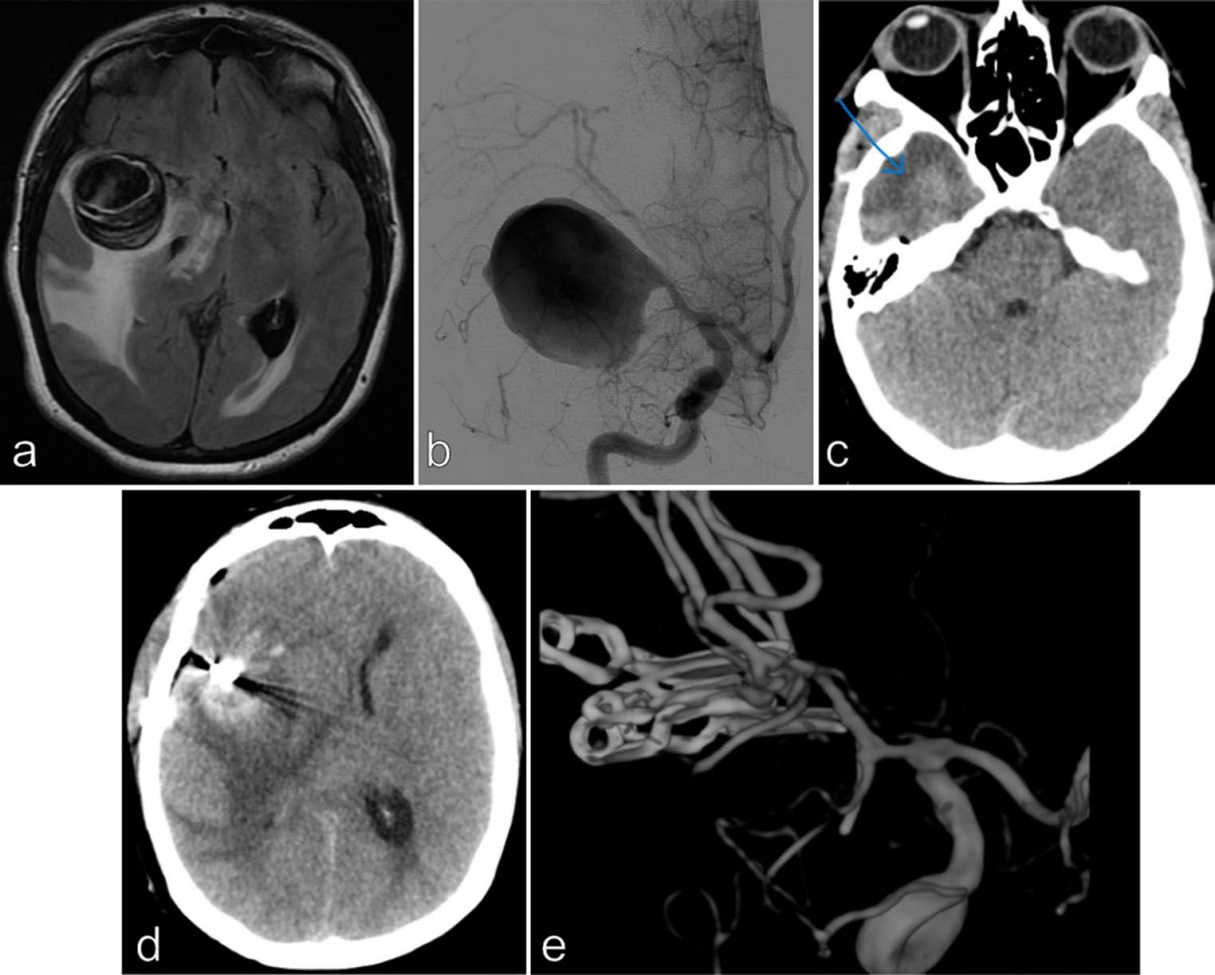
Patient 6, 44 yo female, without any medical history, complained about increasing headache for 1 month. CT scan, MRI/MRA and conventional angiography demonstrated a right giant MCA aneurysm (maximal diameter = 37 mm). A significant peri-aneurysmal edema and intrasaccular thrombus were noted. The midline brain shift was 10 mm (**a** and **b**). Deep hypothermic circulatory arrest/extra-corporeal circulation with vascular reconstruction was performed to exclude the aneurysm from the circulation. A deliberate partial temporal lobe resection **(blue arrow)** was performed at the beginning of the surgical procedure to gain a better exposure of the aneurysm (**c** and **d**). The patient was extubated after 48 h without any neurological deficit. Complete occlusion of the aneurysm was noted (**e**). No neurological sequelae were noted and the mRS score was 1 after 3 months

**Fig. 4:**
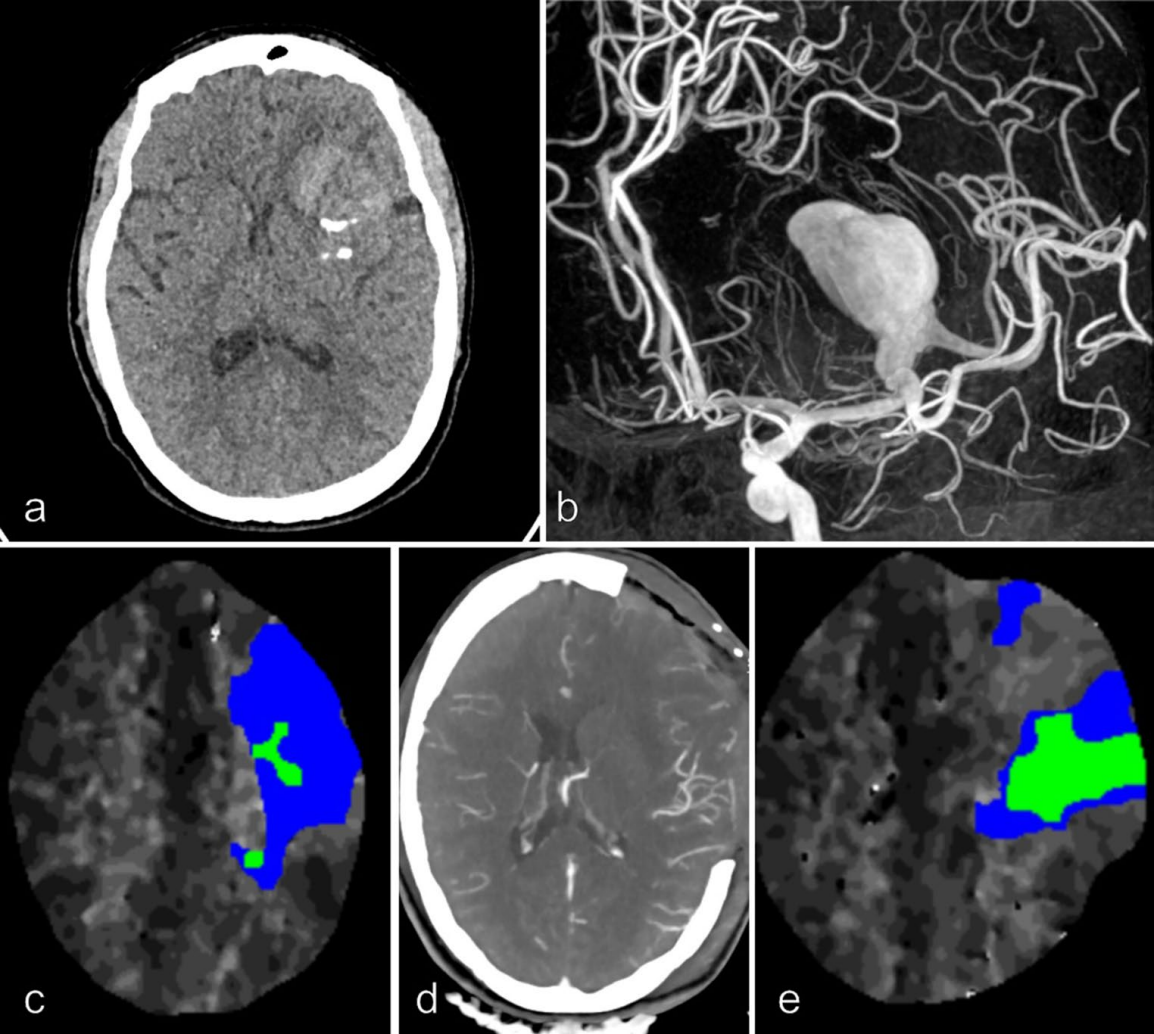
Patient 9, 44 yo female, without any medical history, complained about increasing headache for 3 weeks. CT scan, MRI/MRA and conventional angiography demonstrated a left giant MCA aneurysm (maximal diameter = 50 mm). A moderate peri-aneurysmal edema and intrasaccular thrombus were noted. The midline brain shift was 5 mm (**a** and **b**). A severe hypoperfusion in the left distal MCA territory was noted on the perfusion CT scan (**c**). A distal STA-MCA bypass was performed, followed by aneurysmal trapping and partial resection of the aneurysmal sac. A deliberate decompressive hemicraniectomy was performed at the end of the procedure. The patient was extubated after 24 h without any neurological deficit (**d**). An improvement of the hypoperfusion was recorded on control Perfusion CT scan (**e**). A long-term favorable outcome was recoded (mRS score = 1)

Among the 6 survivors, early postoperative DH was required in 4 patients. A deliberate partial temporal lobe resection was performed in **Patient 6** at the beginning of the surgical procedure and no postoperative DH was required (Fig. 3). The DH was deliberately performed at the end of the surgical procedure in **Patient 9** (Fig. 4). In these 2 last patients, no postoperative neurological deterioration was noted. The mRS score after 3 months was 1 in 2 patients, 2 in 2 patients and 4 in 2 patients. Long term good functional outcome was recorded in 4/9 patients (44%). The late conventional angiography demonstrated a complete occlusion of the aneurysm and an anastomosis patency in all patients. Aneurysm occlusion was noted in each patient at last follow-up.

## Discussion

Severe postoperative outcome was noted in patients with giant MCA aneurysm associated to intracranial hypertension responsible for a midline brain shift. A significant postoperative cerebral edema sometimes associated to ischemic lesions occurred after surgery and led to a DH in most of cases.

### Giant aneurysm treatment

The treatment of giant aneurysms has always been a challenge in the field of neurovascular disease. Nevertheless, many studies mixed patients with a giant aneurysm and patients with a large or complex aneurysm [18, 24], patients with a giant ruptured/unruptured aneurysm, patients with a giant aneurysm of the anterior/posterior circulation [21], patients with a giant aneurysm of the anterior communicating artery or internal carotid artery with patients with a giant aneurysm of the MCA [13, 15]. Therefore, postoperative outcome of these different subgroups of patients remains unclear. Misra [21] reported that 103 out of 134 (76.8%) patients had a good outcome postoperatively. Mortality within 30 days of treatment was 4.47%. Shi [25] proposed a combined approach (coils embolization after bypass protective surgery) for giant MCA aneurysms. Nevertheless, in his limited series of 9 patients, he mixed the patients with ruptured/unruptured aneurysm, with a saccular/fusiform aneurysm, with “only endovascular coiling/combined treatment”, with a mass effect/without a mass effect related to their aneurysm. In our opinion, in patients with intracranial hypertension, endovascular procedure without surgical decompression would not be a safe and an effective treatment. Nevertheless, we still believe that collaboration between endovascular and surgical teams is critical for achieving favorable outcomes in patients with giant MCA aneurysms [5, 6]. Some authors [9, 22, 29] reported predictive factors of poor outcome after treatment of giant intracranial aneurysms: Age, hemorrhage, aneurysms of posterior circulation. No study focused only on the specific subgroup of patients with a giant MCA aneurysm associated to an intracranial hypertension and a significant midline brain shift. Indeed, the treatment of a giant intracranial aneurysm may consider “aneurysmal exclusion” and “vascular tree preservation”. In our specific subgroup of patients, “intracranial hypertension” and “cerebral hypoperfusion” are two further important parameters to consider concerning the treatment of giant MCA aneurysm.

### Intracranial hypertension

The presence of peri-aneurysmal edema is more frequent in patients with giant MCA aneurysm and it was usually associated with the aneurysmal size and the presence of partial thrombosis according to Dengler’s study [7]. Indeed, the intracranial hypertension with the midline brain shift results from the volume of the giant aneurysm and/or the presence of edema. In our series, all patients presented clinical symptoms of intracranial hypertension and significant midline brain shift on radiological assessment. This preoperative intracranial hypertension was always treated by preoperative corticotherapy before surgical procedure even if the edema was absent. Despite this treatment, 7 of 9 patients presented postoperative neurological deterioration with a mean delay of 59 h, related to a significant edematous reaction more or less associated with ischemic lesions. In all patients, cerebral relaxation and vascular patency were obtained at the end of the procedure. The balance of intracranial pressure is precarious in these patients, a small ischemic lesion is enough to conduct to a major and rapid increase of intracranial pressure according to the intracranial pressure-volume curve. Three patients (Patients 1, 4 and 8) were even extubated without neurological deficit before the occurrence of this rapid and severe neurological deterioration. As a postoperative DH was required in 7 of the 9 patients of our specific subgroups, a systematic DH during the surgical procedure (duraplasty, without resting the cranial flap) might be a suitable option. Indeed, as we learned from studies on malignant MCA infarct management [12, 26], it is always better to perform DH preventively, when the patient’s neurological status is still correct rather than in a catastrophic neurological situation. One can argue that there is a potential morbidity related to this decompressive procedure [10] and also to the future cranioplasty [27] (threat to the bypass, infection,…) but in any case, in 7 consecutive patients, an emergency DH with a delayed cranioplasty were necessary in our study. Moreover, perhaps, when the DH is already planned, its morbidity would probably be reduced compared to the morbidity of DH that carried out in emergency situation. This systematic decompressive strategy has already been suggested by some authors in the management of giant aneurysm [2]. A deliberate DH at the end of the surgical procedure was performed in patient 9 with a good early and late functional outcome (Fig. 4). Similarly, a deliberate right partial temporal resection was performed at the beginning of the surgical procedure in Patient 6 with also a good outcome (Fig. 3). She was the second patient who has avoided the postoperative neurological deterioration despite the presence of a significant postoperative edema with a midline deviation. Although resection of healthy brain tissue is not recommended but in this severe disease, it deserves to be discussed.

The alternative strategy would be the placement of subdural/intraparenchymal intracranial pressure (ICP) monitoring probe [30] at the end of the surgical procedure, but, again, a deliberate temporal resection or postoperative DH were necessary in all of our patients anyway, even if the brain did not show swelling during the surgical procedure in all of cases. Perhaps, in case of a deliberate right temporal/frontal resection without a DH at the end of the procedure, the ICP can be suitable. Nevertheless, the limit of the intracranial pressure to perform a postoperative DH remains to be defined, especially in the early postoperative period.

### Hypoperfusion

The hemodynamic impairment caused by the giant aneurysm is under-estimated in the surgical management strategy [14, 28]. The distal hypoperfusion is largely linked to the blood theft and to the damping of the blood flow caused by the giant aneurysm. The mechanical microvascular compression caused by the giant aneurysm is an other parameter to consider in the physiopathology of the hypoperfusion. Indeed, numerous micro-vessels (arteries and veins) might be occluded by the volume of the giant MCA aneurysm and the presence of intracranial hypertension. In our series, 6 patients had a severe hypoperfusion in the MCA zone. In these patients, only low-flow bypass may be proposed. High-flow bypass can conduct to a brain hemorrhage or a reperfusion syndrome. In patient 1, the flow measured in the M3 segments, which originated from the aneurysm was too low compared to the cut flow of the STA branches (5 mL/ minute vs. 25 mL/min). A reperfusion syndrome probably occurred after the anastomosis and has participated to occurrence of the important edematous reaction in this patient. Therefore, the pial natural anastomoses and the collateral natural vascular anastomoses deserve a better evaluation before and during the surgical procedure [3, 4, 16] to define the exact flow required to revascularize the distal zone. For example, in Patient 1, a measurement of the flow in the M3 segments could be done with intraoperative Doppler before and after temporary clipping of the M2 temporal segment (parent artery of the giant aneurysm) during the procedure. With this strategy, the flow provided by distal pial and collateral anastomoses and the required flow needed to revascularize the distal zone could be better evaluated. Perhaps, in some cases, a bypass may not be mandatory. Nevertheless, the aneurysmal sac partial resection may lead to microvessels decompression and, thus, may be responsible for a potential additional flow, which might participate to the reperfusion syndrome. This potential additional flow due to the acute vascular decompression is difficult to be assessed. It appears close to the “Normal Perfusion Pressure breakthrough” and/or the “occlusive hyperhemia” mechanisms occurring sometimes after arteriovenous malformation resection [1]. This mechanism of acute decompression also concerns the veins, which end up thrombosing and worsening the postoperative edema. In fact, the patients with a giant MCA aneurysm associated to an intracranial hypertension have a “Moya-Moya syndrome” associated to a severe intracranial hypertension. In Patient 8, a severe distal hypoperfusion was documented and only distal STA-MCA bypass was performed without exclusion of the giant aneurysm: she developed a rapid and important cerebral edema 10 days after the surgical procedure with a fatal issue. Partial thrombosis of the aneurysm was recorded. The blood flow from the bypass was probably not accurate and disturbed the fragile local hemodynamic balance.

## Conclusion

The giant MCA aneurysms associated to a midline brain shift are challenging for the microsurgical treatment. In our experience, an important postoperative edema reaction occurred in all patients and conducted to a potential DH. We suggest to discuss a systematic DH at the end of the surgical procedure and/or a partial temporal lobe resection at its beginning to reduce the consequences of the edema reaction and to improve the outcome of this subgroup of patients. A better intraoperative assessment of the blood flow might also reduce the occurrence of the reperfusion syndrome.

## Data Availability

No datasets were generated or analysed during the current study.
